# Charting the course of Physiology within the post‐Bologna European higher education area: Insights from Portugal

**DOI:** 10.14814/phy2.15959

**Published:** 2024-03-05

**Authors:** Luis Monteiro Rodrigues, Iris Guerreiro, Vera M. S. Isca, João Gregório

**Affiliations:** ^1^ CBIOS ‐ Research Center for Biosciences and Health Technologies Universidade Lusófona Lisboa Portugal

**Keywords:** discipline characterization, EHEA, physiology education, Portugal and the European higher education area, post‐Bologna Process

## Abstract

The future of physiology has been a recurrent concern for physiologists and Physiological Societies within post‐Bologna Europe and the European Higher Education Area (EHEA). Our paper provides an overview of Physiology teaching and research in Portugal, an EU member state and part of the EHEA. A descriptive study was designed to analyze data publicly available from the National Higher Education Directorate agency (DGES) from September to November 2022 to find all Portuguese syllabi containing at least one discipline related to human Physiology. A detailed database was established, including teaching staff, with a total of 365 courses/degrees and 764 Physiology disciplines. A bibliometric analysis of the identifiable lecturers' scientific production between 2017 and 2022 was made using Web of Science and PUBMED databases. Physiology is part of all health‐related professions. However, universities and technical colleges differ greatly in programs, staff backgrounds, and scientific profiles. Medical schools were found to provide the most complete formation. Noteworthy, the profession of Physiologist has practically no expression within the EHEA, compared with the USA‐UK realities. A better knowledge and understanding of these Physiology modalities in teaching and research within the EHEA will be instrumental to defining a stronger identity for European Physiology in the near future.

## 1 INTRODUCTION

In Western countries, human Physiology has been regarded as a core discipline in medical education, principal in the preparation of highly educated professionals acting in diverse fields, and fundamental in producing advanced knowledge and discovery in related research and innovation (Joyner, 2011; Rees, 1929; Tobin, 2019). In recent years, however, Physiology's relevance has been questioned (Michael, 2006; Naftalin, 2011), and has progressively become a recurrent concern among Physiology organizations worldwide (IUPS, n.d.; Eisner et al., 2013; Gregorio, 2019; Sengupta & Barman, 2018).

The discipline of Physiology gained special recognition in the second half of the 19th century. A wider dispute between the so‐called “vitalism” deeply rooted in Europe and the fast‐expanding fields of physics and exact sciences brought an unusual visibility to these areas in life sciences (Rees, 1929). Johannes Muller became a major personality of this period, rejecting all systems of Physiology that were not founded upon a strict observation of nature and experimental evidence. The publication of the *Handbuch der Physiologie des Menschen*, translated into English as “Elements of Physiology” (Muller & Bally, 2009), is a recognized landmark of this new era for Physiology. This was also the time of the founding of organizations such as the Physiological Society (1876) and the American Physiological Society (APS) (1887) and the creation of the Nobel Prize in Physiology or Medicine, part of the legacy of Alfred Nobel for exceptional scientific achievements in Physiology related to the field of medicine.

During the last 25 years, Physiology has become a reference knowledge domain in human health higher education systems within the European Union (EU) state members and beyond. These systems have been specially shaped by the Bologna Process, which was designed to establish a European Higher Education Area (EHEA) to develop a more globally competitive and attractive academic system within Europe (European Commission/EACEA/Eurydice, 2020). To that purpose, the challenge was to harmonize different systems with different strategic plans, practices, and traditions in order to reach a shared system for academic assessment, including quality assurance. This is the origin of the European Credit Transfer and Accumulation System (ECTS), a central instrument designed to accelerate mutual recognition and academic as professional mobility (European Commission, 2015; European Commission/EACEA/Eurydice, 2020). Various directives were produced in all domains to harmonize syllabus contents and teaching, with credits established as a function of the number of in‐person contact hours dedicated to training, so that 25 teaching contact hours currently correspond to 1 ECTS, complemented by an equal amount (1 ECTS) of autonomous work (European Commission, 2015). Under this framework, multiple, specific regulatory directives were adopted by member countries and health professions (e.g., Medicine, Nursing, Pharmacy, Nutrition) in an effort to give greater parity to professional degrees. In sequence and among others, Physiology became a mandatory component of health profession related programs within the EHEA, existing in a wide variety of modalities since there is no single model of degree programs (European Commission/EACEA/Eurydice, 2020). The Bologna reforms managed to create an EHEA sustained in three–cycle degree programs with different ECTS. The most common first cycles, or Bachelors/Licensure, have 180 ECTS workload programs followed by 120 ECTS credits for the second cycle (Masters). A few professions (especially in health) combined first and second cycle workloads, resulting in 300 ECTS Integrated Masters found in almost all EHEA countries. Cycles corresponding to Doctoral‐PhD studies are also transversal and equally diverse (European Commission/EACEA/Eurydice, 2020). However, different education systems, involving different backgrounds, interpretation, and practices, can challenge implementation and harmonization across members.

We initiated this study primarily to characterize the status of Physiology in Portugal, an EU member‐state and part of the EHEA within the post‐Bologna period. To the best of our knowledge, such analysis has not been made in any of the 29 EU members or within the 49 participating countries of the Bologna Process. This study, initiated during the 2nd International Meeting of the Portuguese Physiological Society (Rodrigues et al., 2023; Rodrigues, Guerreiro, et al., 2023) gathers official information obtained from the public database available at the national Higher Education Directorate agency (DGES–Ministry of Science, Technology and Higher Education). In it, we identified all Higher Education institutions with syllabi including Physiology or Physiology‐related curricular units (CU) in Portugal. After this step, we identified the type of teaching, dedicated hours, and credits for Human Physiology disciplines through a thorough search of all the institutions' webpages. Secondarily, we categorized their principal areas of research interests through the identifiable teaching staff. We expect these results will improve understanding of Physiology teaching and research profiles in a post‐Bologna Process in Portugal and, through this, permit us to obtain greater insights that help to define a more robust and better defined identity for Physiology as a cohesive and essential discipline throughout Europe in a near future.

## 2 METHODS

A descriptive study with a mixed‐methods quantitative and qualitative two‐stage methodology was designed with the goals of (a) identification and characterization of the institutions and curricula including one or more Physiology CU in Portugal, and (b) analysis of the scientific production metrics associated with the respective staff by professional area.

The DGES public database (https://www.dges.gov.pt/) was consulted from September to November 2022 to find all syllabi in the Portuguese higher education system showing at least one CU related to human Physiology. The requirement to include a Physiology CU was either an explicit mention of the word “Physiology,” even if partially combined with other designation, or in its absence, wording in the CU designation related to the entire human body function or functional mechanisms. After identifying institutions, professional areas, and syllabi, each curricular structure, previously confirmed to be in its current legal form, was assessed at the respective public webpage. A database including the higher education institution type (university or technical college), the district, curricular program, academic degree, selected CU total amount of training hours per week, and type of classes (tutorial, laboratorial, other) and credits (ECTS) was established. The database is available upon request to the corresponding author at ZENODO‐https://doi.org/10.5281/zenodo.10609321.

Names of the CU chair/coordinator and other staff were also registered, where available. This initial database comprised a total of 402 different courses/degrees. Physiology CUs in syllabi not related to the human body (e.g., veterinary and related courses, plant, and microbial biology) were excluded. Courses whose degree could not be clearly identified (*n* = 3) and one PhD (*n* = 1) course were also excluded from the final analysis, resulting in a final database of 365 courses and 764 Physiology CUs.

The bibliometric analysis of the lecturers' scientific production began with the selection of a designated coordinator or responsible scholar on the available webpage for any given Physiology CU. When the same scholar was identified in a different CU, an alternative scholar from the staff, if existent, was chosen by the ranking order shown on the website. Through their respective ORCID identifier, a complementary search of the Web of Science and PUBMED databases was then performed to obtain all publication authorships for these members in the five prior years (2017–2022). At ORCID, we manually counted the “Article in indexed journal” publications. We also registered the thematic areas of publication when available in the Web of Science database. All available abstracts were collected for a separate dataset/collection in ZOTERO® (v. 6.0.3, Fairfax, Virginia, USA; available at www.zotero.org), which allowed subsequent in‐depth bibliometric analysis and identification of the most prevalent research topics.

To assist in the bibliometric and network analysis, we exported the Zotero® dataset as a. RIS file and used VOSviewer (v 1.6.19 v 1.6.19, Leiden, The Netherlands), a software tool for scientific mapping, to construct and visualize the authorship and keyword co‐occurrence networks (McAllister et al., 2022). Our aim was to identify relevant clusters of keywords and terms to develop a “picture” of current Physiology research in Portugal. Upon identifying those clusters, we performed a thematic analysis of the words in each cluster to identify common sub‐themes and themes. The thematic analysis regarding the identification of physiological‐related research was assisted by OpenAI's ChatGPT‐3.5 (OpenAI, L.L.C.,77 San Francisco, CA, USA).

## 3 RESULTS AND DISCUSSION

Our first set of data rendered more than 300 health‐related courses (*n* = 365) with 764 Physiology CUs as part of Bachelors/Licensures or Integrated Masters programs offered by technical colleges (57.3%) and universities (42.7%) throughout the country. The geographic distribution was quite uneven, with Lisboa (24.1%) and Porto (22.5%) holding practically half of the available programs offerings. Major cities such as Coimbra (8.2%), Braga (6.3%) Setúbal (4.7%) Leiria (3.8%) and Aveiro, Castelo Branco, and Faro (3.6%) followed. Other district capitals (Bragança, Évora, Guarda, Santarém, Viana do Castelo, Vila Real, and Viseu) showed residual offers (<3%).

**FIGURE 1:**
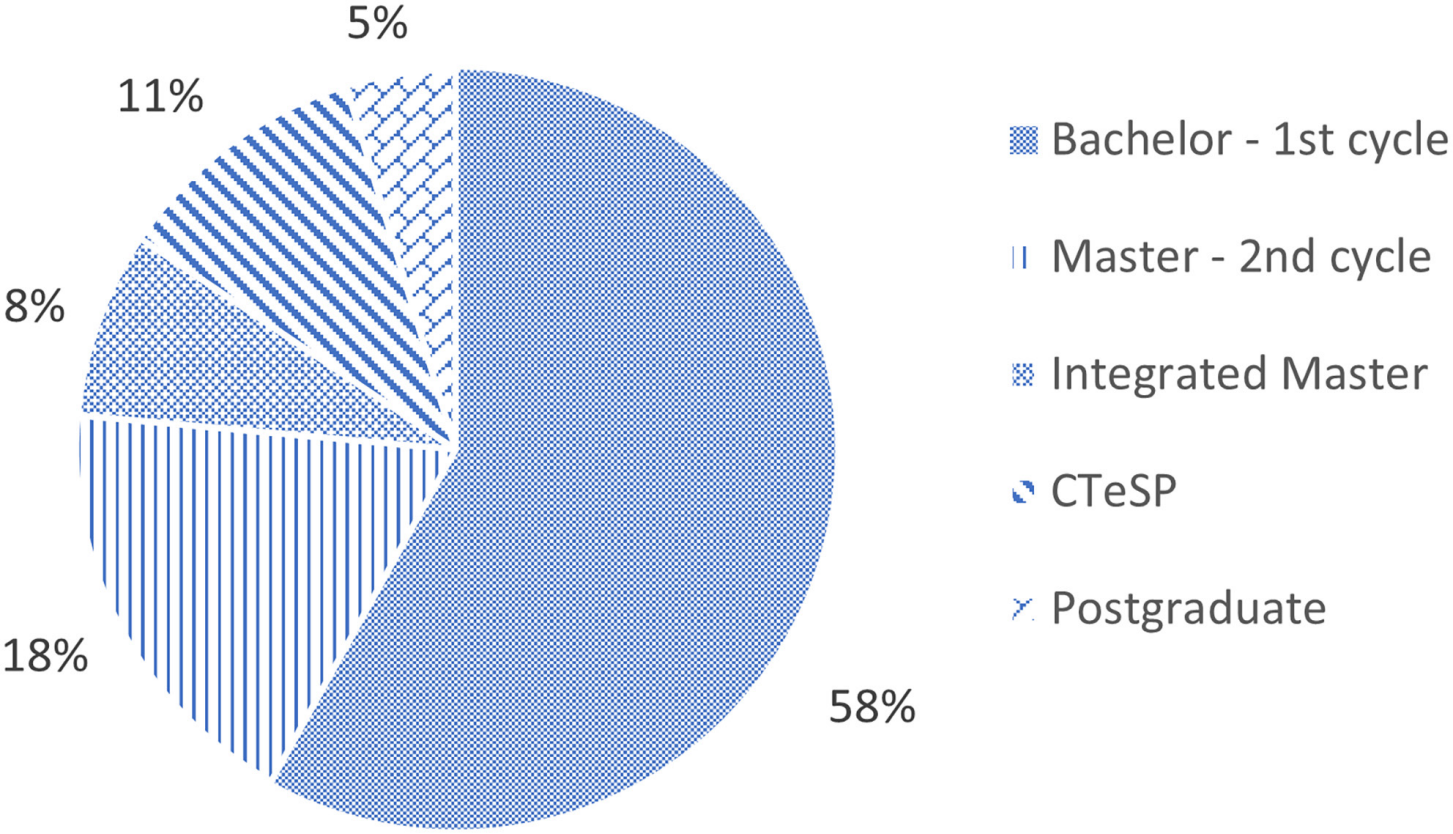
Distribution of academic degrees where Physiology CUs are present.

A clear majority of health‐related educational programs with Physiology CUs (58%) are first cycle Bachelors or Licensure degrees offered at universities and technical colleges (Figure 1). The percentage of Integrated Masters degrees represented by highly differentiated professions (e.g., medicine) is smaller (8%) and exclusively conceded by universities. Professional advanced courses with or without academic degree, involving Physiology represent 23% (18% second cycle Masters and 5% postgraduate no degree involved). CTeSP, freely translated as “higher professional technical program” recently emerging in technical colleges, correspond to 11% of our sample. Being part of the higher education system, these do not offer an academic degree but rather a technical‐professional diploma and, through this, an alternate means to access the higher education system (DGES – CTeSP, n.d.) (Figure 1).

**FIGURE 2:**
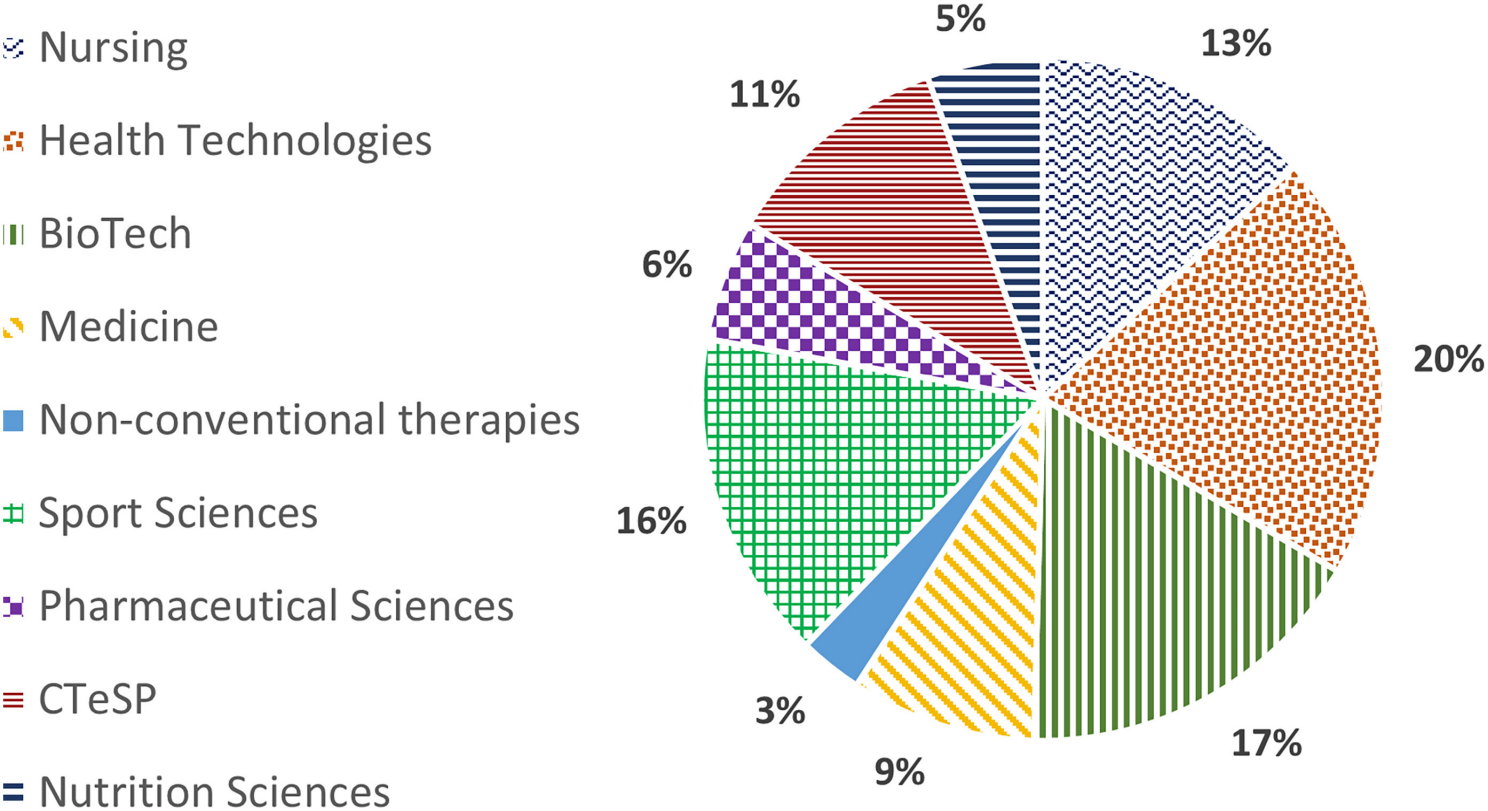
Profession areas encompassing all the different health related educational programs including Physiology CUs (*n* = 365).

These programs were then sorted into nine vocational (professional) areas, as shown in Figure 2, to proceed with the characterization of Physiology in the Portuguese higher education system. Technical colleges are responsible for those first cycle Bachelor/Licensure in Nursing and Health Technologies. The later includes Physiotherapy, Clinical Chemistry, and Clinical Physiology, among others. The majority of these first cycle programs are 3‐year duration, 120 ECTS credits, including specific internships. Exceptions are Nursing and Physiotherapy, each with a 4‐year duration and 240 ECTS credits. More recently, these technical colleges can also offer Nonconventional Therapies first cycle programs (e.g., Acupuncture, Osteopathy) corresponding to Bachelor/Licensure with a three‐year duration, 120 ECTS credits, also including internships, similar to other health technologies programs. Universities also concede first cycle Bachelor/Licensure degrees in health fields such as Nutrition Sciences, Sport Sciences, and other human–related areas including Human Biology, Biochemistry, and Biomedical Engineering, which we gathered under the Biotech denomination. These are three or 4 years in duration and include specific internships. Noteworthy, universities are solely responsible for the Integrated Masters degrees, necessary to access medical (as well as dental and veterinary medicine), with a 6‐year duration, and pharmaceutical professions, with a 5‐year duration corresponding to 360 and 300 ECTS credits, respectively (Figure 2).

Universities and technical colleges share nearly an equivalent number of Physiology CUs. Still, technical colleges show a higher number of these CUs (47%) since nursing, health technologies, nonconventional therapies, and CTeSP coexist together. University programs are typically developed and confined in physically separated institutions with separate facilities and faculty. Here, sport sciences programs seem to bring together the higher representativity of Physiology CUs.

The prevalence of Physiology CUs is particularly noted in the first year of the technical college programs (79.8%) and is often associated with Anatomy (Tables 1 and 2). In universities, Physiology CUs are fairly balanced in the first 2 years, likely corresponding to knowledge advancement (Table 1). In the third year, a very similar distribution is noted in both universities and technical colleges, likely due to the frequent association with pathology and pathophysiology (Tables 1 and 2). Other associations, in particular with alternative denominations not involving the word “Physiology”, such as Fundamentals, Structure, Function‐Functional, Systems or Movement, are also present, especially in university programs (Table 2).

**TABLE 1:** Prevalence of Physiology CUs by program year, in universities, and technical colleges (*n* = 749).

Year	University (%)	Technical colleges (%)	Total (%)
First	148 (47.3)	348 (79.8)	496 (66.2)
Second	127 (40.6)	53 (12.2)	180 (24.0)
Third	31 (9.9)	27 (6.2)	58 (7.7)
foutth	5 (1.6)	8 (1.8)	13 (1.7)
Fifth	2 (0.6)	Not applicable	2 (0.3)

**TABLE 2:** Most common associations of Physiology CUs (*n* = 749).

Alternative denomination and associations	University (%)	Technical college (%)
Anatomy and anatomophysiology	76 (24.3)	209 (47.9)
Neurophysiology and electrophysiology	8 (2.6)	28 (6.4)
Physiopathology and pathophysiology	195 (62.3)	192 (44.0)
Fundamentals, structures, systems	30 (9.6)	5 (1.1)
Movement	4 (1.3)	2 (0.5)

**FIGURE 3:**
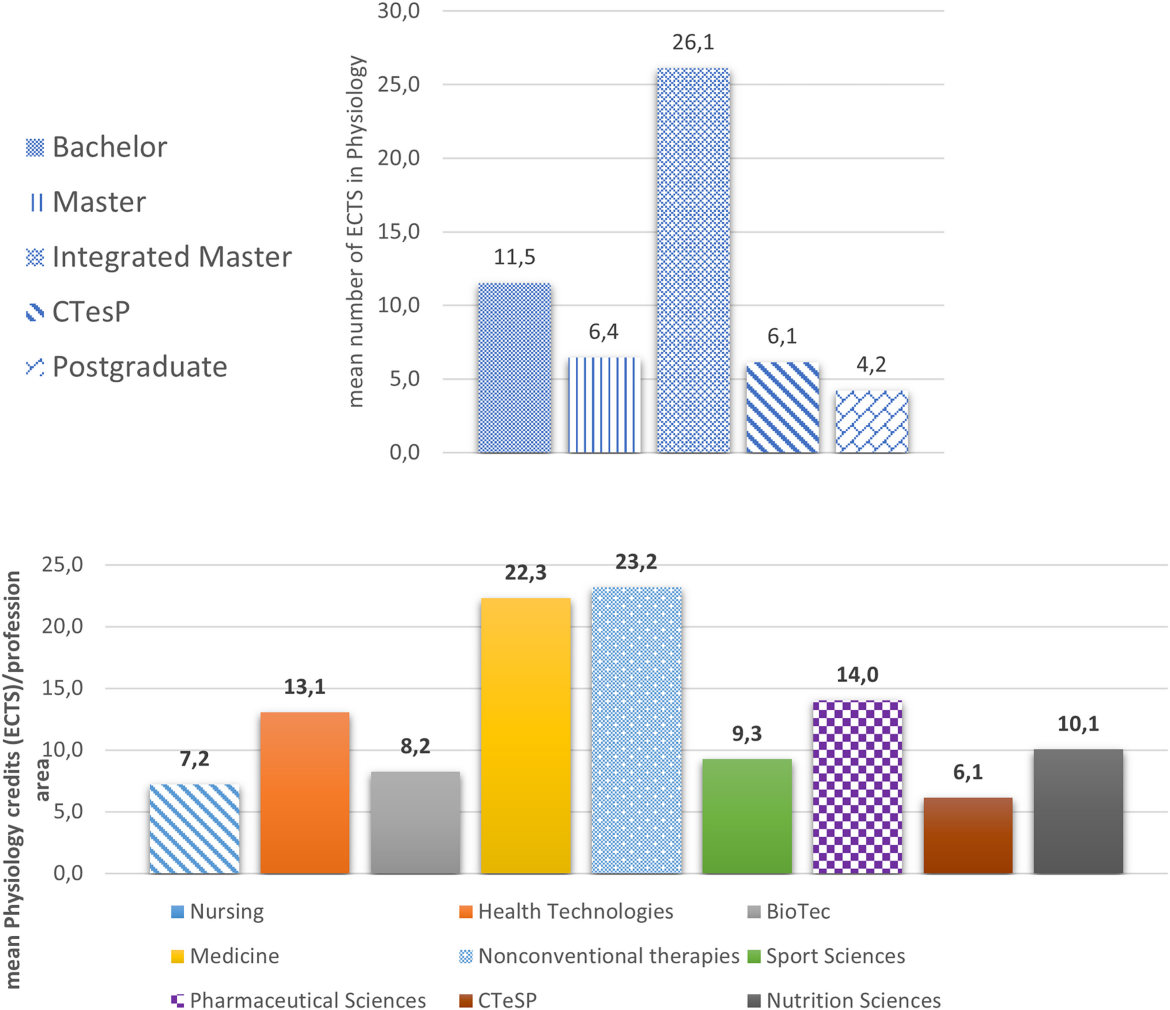
ECTS's (mean) dedicated to Physiology curricular units by academic degree (upper panel) and by professional area (lower panel).

Bachelor/Licensure and Integrated Masters programs usually show the highest number of ECTS credits. The mean credits corresponding to Physiology CUs (Figure 3, upper panel), in proportion to the global number of credits and its distribution per vocational area, were also calculated (Figure 3, lower panel). The number of attributed credits per discipline expresses the relative significance of that CU within that particular semester/year as related to the complete syllabus. As shown in Figure 3 (lower panel), Physiology credits in medicine (Integrated Master) and nonconventional therapies (Bachelor/Licensure) stand out with similar relevance (22.3 and 23.2, respectively) within the respective syllabus structures, far ahead of other profession areas. If this high relevance in medicine is not surprising due to the long history track of Physiology education and research in medical schools, the parallel option of Physiology credits in nonconventional therapies cannot be immediately perceived. These programs, mostly represented by acupuncture (*n* = 3) and osteopathy (*n* = 8), are recent in Portugal (less than 5 years) and administered in technical colleges, where Physiology teaching is also recent. The next vocational area, pharmaceutical sciences, a university Integrated Masters with a 14.0 ECTS for Physiology units, is closely followed by health technologies (13.0) from technical colleges.

**FIGURE 4:**
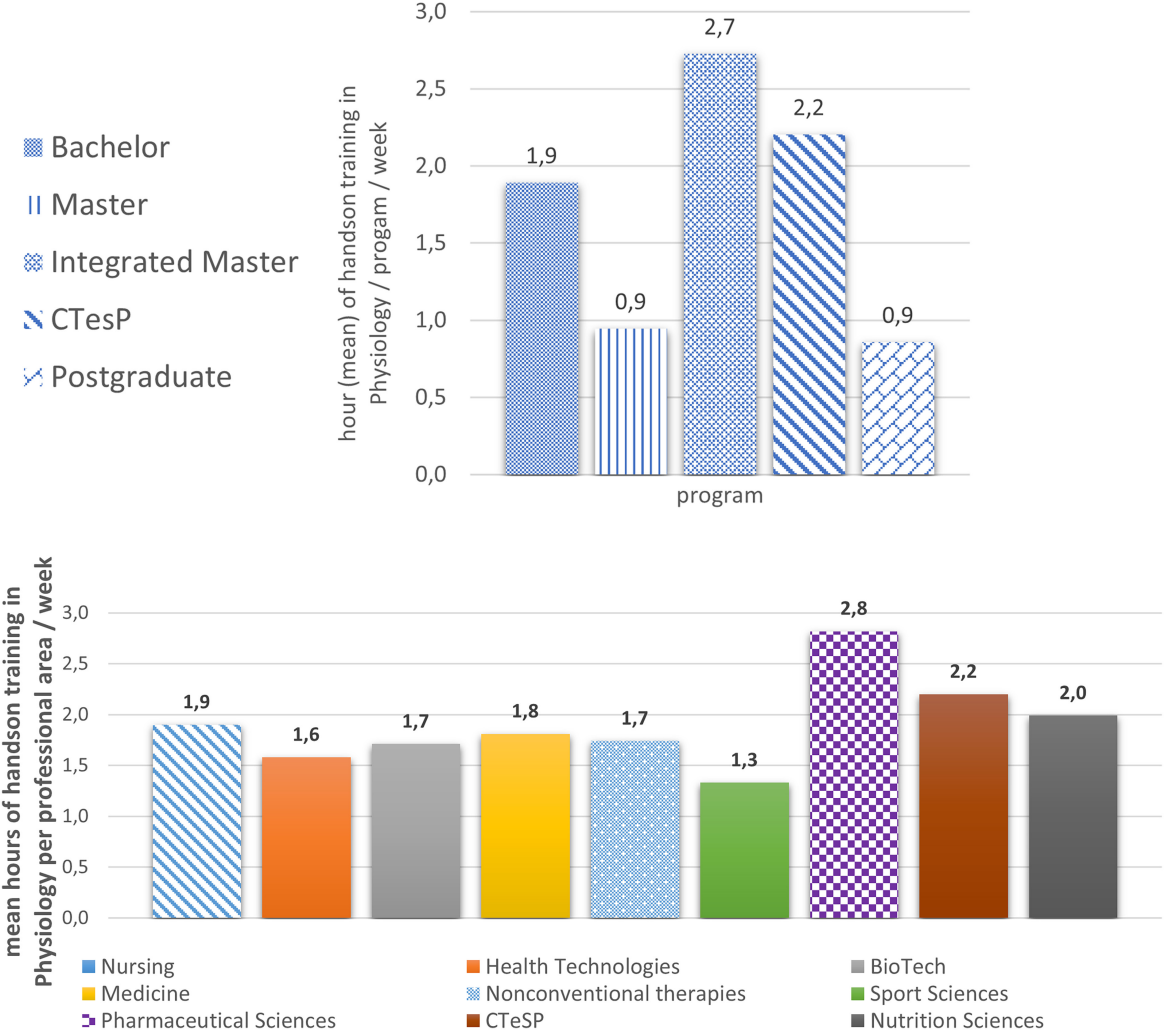
Hands‐on Physiology training per week (mean) by type of program (upper panel) and professional area (lower panel).

To calculate the mean number of hands‐on teaching hours per week, as a measure of the practice/experimental effort dedicated to training in each professional area, we divided the total number of practical teaching hours of Physiology by the number of CUs in each program (Table 3). This calculation showed an unexpected leading position in the university programs for pharmaceutical sciences (2.8), with nutrition sciences (2.0) and medicine (1.8) following, and sports sciences showing the lowest value (1.3). At technical colleges, first cycles nursing, nonconventional therapies, and health technologies were fairly balanced (1.9, 1.7, and 1.6, respectively) (Figure 4).

**TABLE 3:** Mean and median number of Physiology CUs by professional area.

Professional area	Mean	Median	SD
Nursing	1.6	2.0	0.7
Health technologies	2.5	2.0	2.3
Nonconventional therapies	4.6	3.0	3.2
CTeSP	1.2	1.0	0.7
Medicine	3.2	2.0	2.4
Sport sciences	2.0	2.0	1.2
Pharmaceutical sciences	2.6	2.0	0.8
Nutrition sciences	1.9	2.0	0.7
BioTech	1.50	1.00	0.594

We did not compare objectives, competences, and specific contents within these syllabi. However, we will underline that in Portugal, university programs are offered in specialized academies, which are physically independent, in most cases, with proper career staff, some of whom are completely dedicated to research supported by extramural grants. Technical cycles normally coexist in the same space, meaning that the Physiology staff is usually smaller, transversal to all programs, maximizing facilities, programs, and teaching‐learning mechanisms, and instruments with fewer requirements.

We believe that this wide visibility of Physiology is primarily determined by the requirements of the national accreditation agencies. However, keeping in mind that the purpose of our study is not to explain why these similarities or differences exist within the same educational system, we also think that the number and expertise of human resources might also play a role in connecting teaching learning, and research (Borges & Mello‐Carpes, 2014; Crowther, 2017; dos Santos et al., 2021; Granjeiro, 2019) in this discipline, as in others. Thus, in the second stage of this work, we analyzed a convenience sample of Physiology CUs coordinators (chairs) and teaching staff production, to examine their scientific output and identify their main areas of research. The sole inclusion criteria was the inclusion of staff with identifiable ORCID IDs, which provided 196 Physiology Cus, that is, 25.6% of the total Physiology Cus identified in the first stage. Within this subset, only 109 individuals presented a publication track between 2017 and 2022, either articles in indexed publications or conference papers and proceedings. The large majority (2/3) belonged to university curricular programs, led by Medicine (~50%), while the remaining 1/3 belonged to technical colleges, primarily within Nursing and Health Technologies (Table 4).

**TABLE 4:** Distribution of the selected curricular units with staff information by professional area.

Professional area	Selected physiology CUs	Physiology CUs staff with publication track record between 2017 and 2022
Medicine	51	26
Sport sciences	34	23
Health technologies	32	15
Nursing	24	17
Pharmaceutical sciences	20	11
Biotech	16	10
Nutrition sciences	12	5
Nonconventional therapies	7	2
Total	196	109

Further analysis showed that between 2017 and 2022, almost 40% of this staff produced between 0 and 5 indexed publications in Web of Science or PUBMED detected by ORCID as “Article in indexed journal,” and that 63.3% of these staff published no more than 15 papers within this period. A minority of 15.6% had more than 30 indexed publications in the same period (Table 5).

**TABLE 5:** Distribution of the number of publications of the sample selected staff in indexed journals for the period 2017–2022.

Publication interval	Number of authors (%)
0	22 (20.2)
1–5	21 (19.2)
6–10	15 (13.8)
11–15	11 (10.1)
16–20	8 (7.3)
21–25	7 (6.4)
26–30	8 (7.3)
More than 30	17 (15.6)

Considering the crude scientific output per area, we found that the highest output belongs to Medicine (517), closely followed by Sports Sciences (454) (Table 6). The long tradition of Physiology teaching in research in medical schools as in sports sciences schools gradually transformed these institutions to become principal schools of Physiology (Borges & Mello‐Carpes, 2014; Crowther, 2017; Gregorio, 2019; Joyner, 2011; Rodrigues, Guerreiro, et al., 2023; Sengupta & Barman, 2018). That likely suggests the proximity between the observed outputs between these areas. Other areas follow closely, confirming the interesting production from Nursing and Health Technologies (Table 5).

**TABLE 6:** Number of publications, average per scholar, and mean H‐Index per area.

Professional area	Total indexed publications	Average per faculty member (SD)	Average H‐index (SD)
Medicine	517	19.9 (21.3)	13.9 (12.0)
Sport sciences	454	19.7 (21.2)	8.8 (6.3)
Health technologies	238	15.9 (24.1)	8.67 (9.2)
Pharmaceutical sciences	174	15.8 (17.9)	12.6 (8.5)
Nutrition sciences	144	28.8 (26.5)	25.6 (9.9)
Nursing	112	6.6 (10.9)	1.06 (1.6)
Biotech	112	11.2 (7.9)	17.6 (9.3)
Nonconventional therapies	10	5.0 (7.1)	0.5 (0.7)

Results show a wide dispersion in these areas (high standard deviation), which signifies that the considerable scientific output likely results from a smaller number of authors with different outputs. We can also imagine that these principal schools have more resources (human and material) and ongoing funded projects, which facilitates co‐authorship and more expressive outputs.

It should also be noted that nutrition sciences and biotech areas include authors with the highest impact, as measured by their respective H‐index.

**FIGURE 5:**
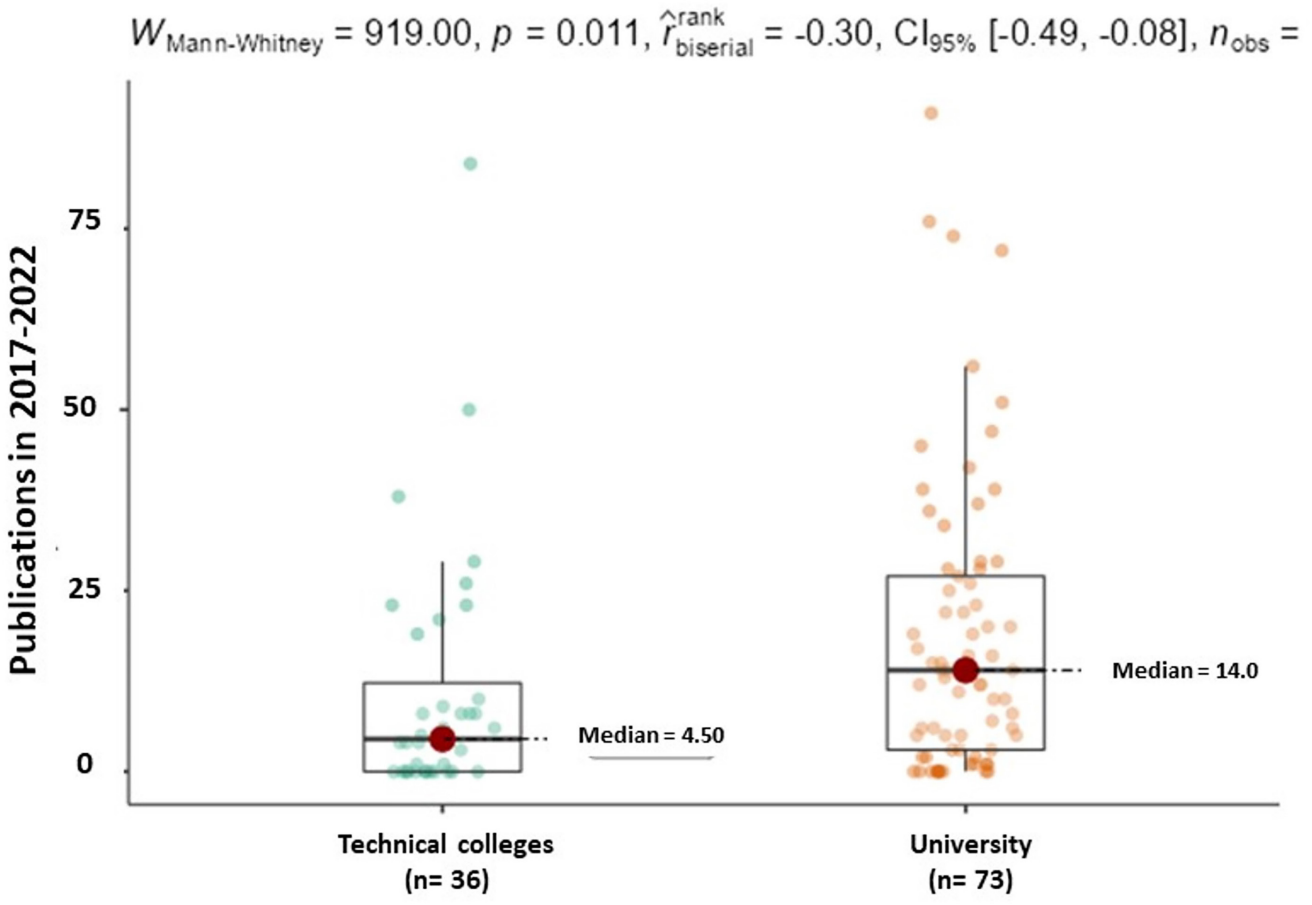
Number of identifiable publications from Physiology staff by type of institution (*n* = 109).

In any case, looking into the differences by type of institution (Figure 5), it is clear that university authors have a significantly higher scientific output (p = 0.011) compared with their corresponding colleagues within technical colleges.

Sixty‐five different journal thematic areas were identified among the publications. Only five authors, mostly those from Sport Sciences, Pharmaceutical Sciences, and Medicine, published in Physiology journals (4.6%)—that is, journals with the words “physiology/physiological” in their title (Table 7). The most relevant thematic area is “Medicine (general or internal)” representing 37.6% of publications within this sample. It should be noted that Physiology‐Pharmacology/Toxicology journals areas are frequently related, and were selected by 22 authors (20.2%).

**TABLE 7:** Distribution of scholars' publications per thematic area.

Journal thematic area	% of publishing teachers	Top 3 main vocational areas
Physiology	4.6%	Sport sciences Pharmaceutical sciences Medicine
Pharmacology and toxicology	15.6%	Pharmaceutical sciences Medicine Biotech
Nursing	14.7%	Nursing Sport sciences Biotech
Neurosciences and Neurology	14.7%	Health technologies Medicine Biotech
Microbiology and immunology	14.7%	Medicine Pharmaceutical sciences Nutrition sciences
Medicine (general, internal)	37.6%	Medicine Sport sciences Biotech
Genetics, molecular, and hereditary biology	22.0%	Medicine Biotech Nutrition sciences
Environmental and health sciences	14.7%	Sport sciences Health technologies Pharmaceutical sciences
Chemistry and chemical engineering	14.7%	Sport sciences Biotech Medicine
Biomedical engineering and engineering (general)	13.8%	Medicine Sport sciences Biotech
Biochemistry	22.9%	Pharmaceutical sciences Medicine Health technologies

**FIGURE 6:**
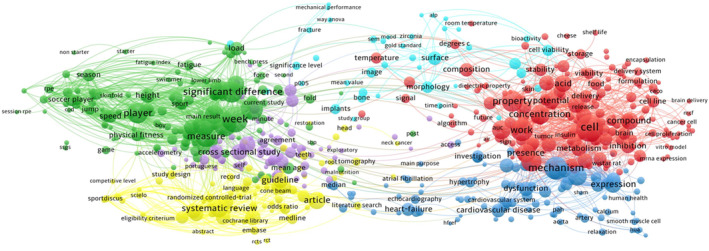
Network visualization map of co‐occurrence of terms and keywords (Minimal occurrences = 7, Normalization method–association; Merge small clusters–yes). The red cluster gathers keywords and terms mostly related to active substances or interventions that impact living systems (from the cell to the experimental animal), both in healthy as in non‐healthy conditions. The green cluster contains most of the terms related to activeness performance and respective markers, also including the associated study methodology and indicator terms. The dark‐blue Cluster closely touches the Red Cluster involving mechanisms of action, expression, and pathways, where cardiovascular indicators are also frequently referred. The yellow cluster presents terms related to experimental design and methodologies, mainly systematic reviews. Finally, the purple cluster seems to complete the “methodology” clusters (green and yellow), while the light‐blue cluster seems to include most of the specific terms related to dental surgery closer to the red cluster.

The analysis of abstracts and keywords allowed the identification of 29,348 single terms. To perform the co‐occurrence cluster analysis, we chose to present the visualization of terms that co‐occurred at least seven times, yielding a total of 1060 terms. From these, the 60% most relevant (636) were organized in six clusters (Figure 6). VOSviewer allows the selection of the number of terms to include in the cluster design. Our criteria aimed to produce the most detailed clusters possible without negatively impacting the clarity of the resulting figure. After the clustering provided by VOSviewer, we performed thematic analysis of the words in each cluster, aiming to identify common themes and sub‐themes (Table 8).

**TABLE 8:** Thematic analysis of the six clusters: main research topics and keywords across clusters.

Cluster	Theme	Subtheme	Associated terms and keywords (examples)
Red	Biomedical research and innovations	Medical research & therapies: Advances in disease treatment	Cancer; treatment; drugs; epilepsy; pharmacokinetic; neurodegenerative disease
		Biological dynamics: Cellular metabolism & growth	Metabolism; growth; compound; production; cell; potential
		Molecular understanding: Genetic expression & compounds	Gene; expression; potential; compound; alteration; work
		Experimental techniques: Cutting‐edge methodologies.	Assays; intranasal administration; microwave imaging; simulation; Western blot; detection
		Delivery and administration: Drug delivery, nanoparticles, blood–brain barrier.	Delivery; administration; compound; presence; potential; brain
Green	Exercise physiology and performance metrics	Diverse exercise methods: Varied workout techniques	Aerobic exercise; strength training; high‐intensity interval training (HIIT); sprint; agility
		Physical metrics & anthropometry: Body measurements & strength	Body mass index (BMI); muscle strength; height; anthropometry; body composition
		Physiological indicators: Heart rate, BP & variability	Heart rate; heart rate variability (HRV); blood pressure; ejection fraction; peak torque
		Performance metrics: Agility, jump & endurance	Agility; jump; endurance; speed; repetition maximum (RM)
		Research methods: Study design & statistical analysis	Cross‐sectional study; ANOVA; exploratory research; statistical significance; T test
Dark blue	Cardiovascular health, function, and pathology	Cardiovascular system: Heart health & function	CVD; heart; atherosclerosis; cardiac function; arterial stiffness
		Cardiac function & dysfunction: Heart performance & issues	Heart failure; ejection fraction; arrhythmias; diastolic dysfunction; systolic blood pressure
		Molecular mechanisms and expression	Gene expression; signaling pathways; molecular mechanism; pathway; regulation
		Physiological regulation and homeostasis	Endocrine; metabolism; oxygen; hormonal; homeostasis
		Pathological processes and conditions	Atherosclerosis; hypertension; inflammation; coronary artery disease; myocardial infarction
Yellow	Research literature and methodology	Literature exploration: Articles, databases & references	Articles; databases; Cochrane Library; PubMed; reference
		Publication & reporting: Peer reviews & PRISMA.	Peer; PRISMA; peer‐reviewed journal; title; article
		Research design: Study methods & analysis	Survey; ANOVA; statistical significance; study design; analysis techniques
		Instrumentation & data collection: Questionnaires & tools.	Questionnaire; interview; validity; instrument; measurement
		Demographics & context: Language, population & region.	Brazil; Spanish; Portuguese; population; university
Purple	Health assessment and methodology	Health and well‐being: Mental health, activity & pandemic impact	Mental health; physical activity level; sedentary behavior; pandemic; QoL
		Physical assessment: Validity, reliability & assessment tools	Reliability; validity; assessment; measurement; assessment tools
		Research design & analysis: Study methods & statistical techniques	Research methods; study design; statistical analysis; data collection; research techniques
		Research instruments and tools: Questionnaires, surveys & interviews	Questionnaires; surveys; instruments; tools; assessment
		Demographics and context: Population, language, setting	Language; population; region; university; student
Light blue	Biomedical materials and analysis	Biomedical research and analysis: Bioactivity, cell viability, SEM.	Bioactivity; cell viability; materials; analysis; research
		Innovative materials: Dental implants, titanium & bone health	Dental implant; titanium; implants; bone; material
		Analytical methods: ANOVA, reliability & significance	ANOVA; reliability; analysis; research; statistical significance
		Biological exploration: Osseointegration & bone processes	Osseointegration; bone formation; fracture healing; biological processes; bone health
		Measurement and evaluation: Mechanical properties, surface assessment.	Stability; formulation; property; exposure; concentration; compound

This simple analysis, restrained to the information available in our convenience sample, seems to complement the observations produced regarding Tables 6 and 7. In fact, the majority of these lecturers' research interests appear to be concentrated into two wide tiers, which we designated as Medical Physiology and Lifestyle Physiology. The Medical Physiology tier involves the Red, Dark‐ and Light‐Blue Clusters, representing physiological and patho‐physiological research interests, with a suggested cardiovascular research prevalence. The Green Cluster is the basis of the Lifestyle Physiology tier, with interests primarily dedicated to sport and exercise physiology, but also showing some new thematics such as body composition, nutrition, and well‐being. Each of these two tiers involves professionals from different areas, mainly from university centers and research units. Physiology research no longer seems exclusive to medical background researchers but it appears that a specific training history in the area is needed to support a “Physiological capacitation” or way of thinking.

Some limitations should be recognized in our approach:

- We did not access syllabi built‐in competencies, hands‐on contents, and teaching‐learning methodologies that might relate to the attributed ECTS credits in each institution;
- We did not assess the staff background and their physiology‐related differentiation;
- We did not make any type of institutional quality assessment for those Physiology CUs;
- We did not measure the relative perceptions about the importance of Physiology within the teaching‐learning community (faculty and student) at the university and technical colleges, as recently recommended (Raif Gregorio Nasre‐Nasser, 2022)

Nevertheless, we expect this exploratory study might promote other similar approaches for a better knowledge of Physiology representation in Europe, which is crucial to define its character and future progress.

## 4 CONCLUSION

Our study could successfully identify all syllabi in Portugal where the Physiology discipline is being taught. A few conclusions can be drawn:

1. Physiology is closely present in universities and technical colleges programs (mainly in the first and second cycles), and also similar in terms of attributed credits and class type; nevertheless, some differences emerge:

- in universities, where Integrated Masters like medicine or pharmaceutical sciences are administered, Physiology or Physiology‐related CUs are sequentially present from year one until late in the course, likely reflecting a progression in advanced knowledge and its application; in some courses, the term “physiology” has been replaced by other designations but the purpose remains;
- in technical colleges, Physiology is prevalent in year one, likely due to its common association with Anatomy, being apparently assumed as a mandatory component of the basic training;
- universities and technical colleges greatly differ in the hands‐on component, which might be related to the different structural organization of these institutions and likely reflect the differences between facilities, available teaching‐learning instruments, and staff preparation;

2 Physiology research exists in the faculty identified within the publicly available sample, not without multiple disproportions:

- the science profile of this academic population seems very asymmetric
- the scientific production of the majority of these lecturers in the last 5 years is scarce or nonexistent;
- significant differences between university and technical colleges' scientific production in these areas are clear; staff with relevant production are affiliated with reference university centers or research units;
- Medical Physiology and Lifestyle Physiology seem to be the two major areas of scientific production, with these researchers' preferences focusing on biomedical research and innovation, cardiovascular function, sport performance and well‐being;

It is likely that Physiology is similarly represented in other EHEA countries as in Portugal. Nevertheless, our findings do not raise any immediate concerns regarding Physiology as a discipline at present, in line with other similar views (Barman et al., 2013; Rodrigues, Gregório, & Wehrwein, 2023). However its presentation diversity and the lack of a recognized professional status, as it exists in the US and in UK countries, suggests that these communities, national and European, must be strengthened. We believe the European Federation of Physiological Societies (FEPS) and local physiology organizations are key to facilitating this process through articulated initiatives aimed to improve physiology knowledge transfer within academia and to a broader society. In both teaching and research, Physiology needs to be recognized and supported to keep its distinctiveness as an integrated science of life needed to shape the approach and rational applied to problems involving our understanding of health and disease and, for those reasons, as essential in human health education and research.

## FUNDING INFORMATION

This study received no funding.

## CONFLICT OF INTEREST STATEMENT

Authors declare no conflict of interest.

## 5 ETHICS STATEMENT

Not applicable.

## Data Availability

Authors confirm that all data reported and used in this study are publicly and referred through a proper citation.
